# When big data initiatives meet: Data sharing between THANADOS and IsoArcH for early medieval cemeteries in Austria

**DOI:** 10.1016/j.dib.2023.109250

**Published:** 2023-05-31

**Authors:** Nina Richards, Stefan Eichert, Sabine Ladstätter, Christina Cheung, Michael P. Richards, Kévin Salesse

**Affiliations:** a Austrian Centre for Digital Humanities and Cultural Heritage, Austrian Academy of Sciences, Bäckergasse 13, 1010 Vienna, Austria; b Department of Prehistory, Natural History Museum Vienna, Burgring 7, 1010 Vienna, Austria; c Austrian Archaeological Institute, Austrian Academy of Sciences, Franz Klein Gasse 1, 1190 Vienna, Austria; d Department of Anthropology, Chinese University of Hong Kong, Shatin, New Territories, Hong Kong; e Department of Archaeology, Simon Fraser University, Education Building 9635, 8888 University Drive, Burnaby, B.C. V5A 1S6, Canada; f Department of Anthropology, Faculty of Science, Masaryk University, Kotlářská 2, 611 37 Brno, Czech Republic

**Keywords:** Stable isotope analysis, Collagen, Carbon, Nitrogen, Sulfur, Paleodiet, Medieval period, Central Europe

## Abstract

This paper reports carbon, nitrogen, and sulfur stable isotope data obtained from bone collagen of humans from the early medieval cemeteries of Hemmaberg/Gora svete Heme and Oberleiserberg located in Austria. The Hemmaberg/Gora svete Heme cemetery, dating from the 8^th^ to the 11^th^ century, comprises 29 graves, from which 15 individuals were analyzed. The Oberleiserberg cemetery, established in the first half of the 11^th^ century, includes 71 graves as well as several incidental finds of human bones, from which 75 samples were analyzed. Both cemeteries show comparable δ^13^C data (mean for Oberleiserberg: –17.5 ± 1.2 ‰, 1σ; mean for Hemmaberg: –16.4 ± 1.6‰, 1σ). However, the δ^15^N values of individuals from Oberleiserberg (mean: +10.4 ± 1.5‰, 1σ) are slightly higher than those of individuals from Hemmaberg/Gora svete Heme (mean: +8.8 ± 1.1‰,1σ). The δ^34^S values were only obtained on the individuals from Oberleiserberg, and show a mean value of –0.9 ± 2.0 ‰ (1σ).

Beyond the isotopic data presented in this article, we lay the foundations for cooperation between the IsoArcH database (https://isoarch.eu) [1] and the THANADOS (https://thanados.net) [2] project. While IsoArcH primarily stores isotope-related datasets for bioarchaeology, THANADOS stores data on archaeologically and anthropologically researched burials. Moving forward, IsoArcH and THANADOS plan to work closely together to integrate their databases. This collaboration presents a promising opportunity for both projects to pool their resources and knowledge, offering a wealth of information for researchers and the general public who are interested in anthropology and archaeology.

**Specifications Table**

**Table:** 

Subject	Archaeology Anthropology
Specific subject area	Stable isotope analysis Collagen Carbon Nitrogen Sulfur Paleodiet Medieval period Central Europe
Type of data	Table Figure
How the data were acquired	Information on the individuals was acquired by anthropological analyses conducted by Nina Richards. The following methods were used: Sex estimation: Ferembach et al. (1979) Age estimation (subadults): Ubelaker (1978), Ferembach et al. (1979), Schaefer et al. (2008), Hunger and Leopold (1978) Age estimation (adults): Nemeskéri et al. (1960), Todd (1920), Szilvássy (1988), Miles (1963), Lovejoy (1985), Schultz (1988) For the Oberleiserberg samples, collagen extraction was carried out in the Isotope Laboratory at the Department of Archaeology from Simon Fraser University (Burnaby, Canada), and stable isotope measurements were performed at IsoAnalytical Limited (Crewe, United Kingdom; using a Isotope abundance mass spectrometer with coupled elemental analyzer (Elemental Analysis - Isotope Ratio Mass Spectrometry, EA-IRMS)). Extraction of collagen was performed according to the specifications in Richards and Hedges [3], supplemented by additional ultrafiltration of the sample material as recommended by Brown et al. [4]. Hemmaberg/Gora svete Heme samples were prepared and analyzed at Poznan Radiocarbon Laboratory (Poznan, Poland; using a Flash Elemental Analyzer 1112 (ThermoScientific), connected to a continuous flow inlet of a MAT 253 gas source mass spectrometer (Thermo Scientific) Collagen was extracted following Longin [5] and Piotrowska and Goslar [6]).
Data format	Raw
Description of data collection	Oberleiserberg: Samples from 75 individuals were examined, of which 70 samples produced collagen of good quality. These included δ^13^C, δ^15^N, and δ^34^S data on 47 subadults and 23 adults. Hemmaberg/Gora svete Heme: The sample includes five adults as well as ten subadults. While all adults but burial 6 were included, subadults were chosen due to location or pathological conditions (primarily vitamin C deficiency). δ^13^C and δ^15^N data were obtained for these individuals.
Data source location	Institutions: The human remains of the Hemmaberg/Gora svete Heme cemetery are curated by the Austrian Archaeological Institute Vienna (Austrian Academy of Sciences); the human remains of the Oberleiserberg cemetery are curated at the Department of Prehistory and Historical Archaeology (University of Vienna) as well as the Department of Anthropology (Naturhistorisches Museum Vienna). City/Town/Region: Vienna/Globasnitz/Oberleis Country: Austria Latitude and longitude for collected samples: Hemmaberg/Gora svete Heme: 46.54531°N, 14.70562°E (WGS84); Oberleiserberg: 48.56667°N, 16.36667°E (WGS84).
Data accessibility	Repository 1: THANADOS (https://thanados.net) [2] Repository 2: IsoArcH (https://isoarch.eu/) [1] DOI of the dataset: 10.48530/isoarch.2021.017 Direct URL of the dataset: https://doi.org/10.48530/isoarch.2021.017 Data is available under the Creative Commons BY-NC-SA 4.0 license.

## Value of the Data

**Fig. 1:**
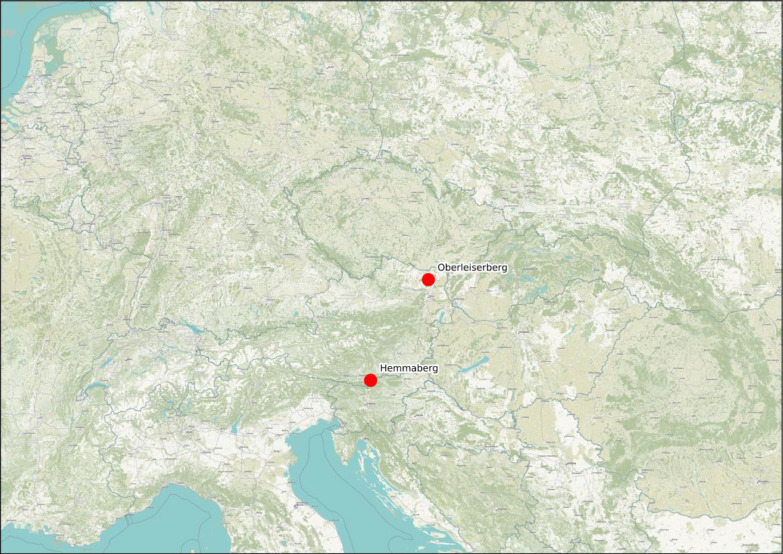
Location of the two sites included in this article. Map: Stefan Eichert, background map: © OpenStreetMap contributors, Tiles style by Humanitarian OpenStreetMap Team hosted by OpenStreetMap France.
• The significance of the results lies in the substantial dataset size, which includes 15 individuals from the Hemmaberg/Gora svete Heme cemetery and 75 individuals from the Oberleiserberg cemetery. In total, 85 individuals provided well-preserved collagen samples. This large sample size allows for in-depth comparisons by other researchers.
• The two burial grounds, Hemmaberg/Gora svete Heme and Oberleiserberg, exhibit an unusual concentration of children, with 56.7% and 68.8%, respectively. This is atypical for the region and era, and thus, they offer valuable comparative information for investigating the subadult population and their dietary habits during medieval times.
• The two datasets offer a unique perspective into the everyday lives of the two medieval communities, primarily through their dietary habits, which cannot be attained through traditional osteological approaches.
• The paper marks the commencement of a joint endeavor between the open-access initiatives THANADOS [2] and IsoArcH [1] to improve the interoperability and knowledge management of their respective databases.

• The data article showcases the results of stable carbon, nitrogen, and sulfur isotope analysis performed on human bone collagen samples from individuals buried in the cemeteries of Hemmaberg/Gora svete (Carinthia, Austria) and Oberleiserberg (Lower Austria, Austria) (Fig. 1). Published isotope studies on early-medieval sites in present-day Austria are scarce.

## 1 Objective

The present data set comprises early medieval isotope data from present-day Austria. So far, only few other datasets of that region and time frame have been investigated and published. This is surprising, since an evaluation can give an important contribution to the habits of populations, which today can only be recorded archaeologically.

Furthermore, the two datasets serve as a basis for a cooperation between the projects THANADOS [2] and IsoArcH [1].

## 2 Data Description

The dataset consists of samples collected from 90 human individuals, of which 15 originate from the Hemmaberg/Gora svete Heme cemetery and 75 come from the Oberleiserberg cemetery. Most of the Hemmaberg/Gora svete Heme graves date to the 8^th^-11^th^ century, except for one fetus or neonate (grave 16) that dates to the 14^th^-16^th^ century [2]. Oberleiserberg cemetery contains graves from the first half of the 11th century. Well-preserved collagen samples were obtained from 15 individuals at the Hemmaberg/Gora svete Heme cemetery and 70 individuals at the Oberleiserberg cemetery (n_total_ = 85) [2].

The Oberleiserberg collection comprises 47 subadults and 23 adults (Table 1). Among the adults, nine were females and ten were males. No sex was determined for two of the adults.

**Table 1:** Age-at-death estimations for analyzed individuals from Hemmaberg/Gora svete Heme and Oberleiserberg.

	Age Category	Age-at-death	Hemmaberg/Gora svete Heme	Oberleiser- berg	Total
Subadults			**10**	**47**	**57**
	Fetus/Neonate	Before birth to 3 months	1	-	
	Neonate	Birth to 3 months	-	7	
	Infans 1	3 months to 6.9 years	7	26	
	Infans 1/2	5 to 11.9 years	-	3	
	Infans 2	7 to 13.9 years	1	6	
	Infans2/Juvenile	10 to 15.9 years	1	2	
	Juvenile	14 to 19.9 years	-	2	
	Child	0 to 13.9 years	-	1	
Adults			**5**	**23**	**28**
	Adult	20 to 39.9 years	3	12	
	Adult/Mature	30 to 49.9 years	1	3	
	Mature	40 to 59.9 years	1	4	
	Grown-up	20 years and older	-	4	
Total			**15**	**70**	**85**

The Hemmaberg/Gora svete Heme sample is composed of five adults and eleven subadults (Table 1). The individual from grave 16 died as a fetus or shortly after birth. However, it does not belong to the early medieval burial community, as it was buried in the 14^th^–16^th^ century. The child was buried without grave goods, but radiocarbon dating results are available on THANADOS [2] - clearly shows a different time frame than the other burials, It is the only burial of this period located on the mountain.

**Fig. 2:**
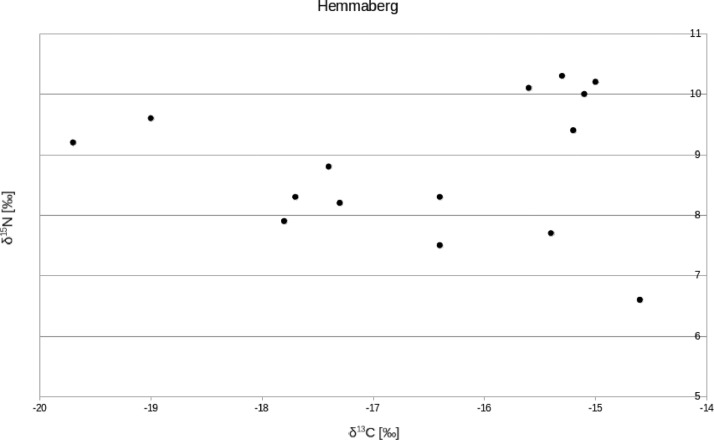
Biplot of δ^13^C and δ^15^N values measured on bone collagen from individuals buried in the Hemmaberg/Gora svete Heme cemetery.

The Hemmaberg/Gora svete Heme individuals have a mean δ^13^C value of –16.4 ± 1.6‰ (1σ) and a mean δ^15^N value of +8.8 ± 1.1‰ (1σ). For adults, the mean δ^13^C value is –17.1 ± 1.4‰ (1σ) and the mean δ^15^N value +8.8 ± 0.7‰ (1σ) (females: –17.6 ± 1.3‰ and +8.7 ± 0.8‰, respectively; males: –16.5 ± 1.8‰ and +8.9 ± 0.8‰, respectively). For subadults, the mean δ^13^C value is –16.0 ± 1.6‰ (1σ) and the mean δ^15^N value is +8.8 ± 1.3‰ (1σ) (Fig. 2).

**Fig. 3:**
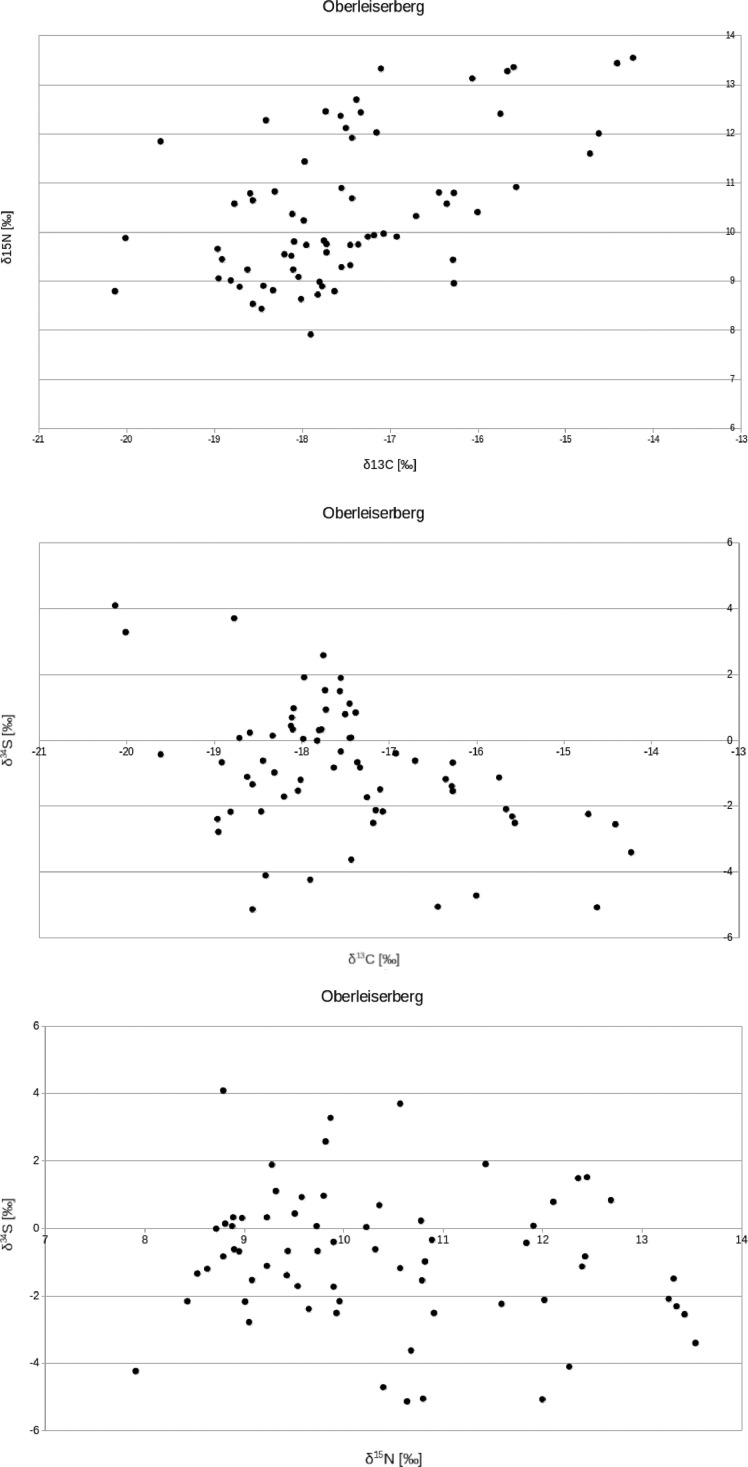
Biplots of (A) δ^13^C and δ^15^N values, (B) δ^13^C and δ^34^S values, and (C) δ^15^N and δ^34^S values measured on bone collagen from individuals buried in the Oberleiserberg cemetery.

**Fig. 4:**
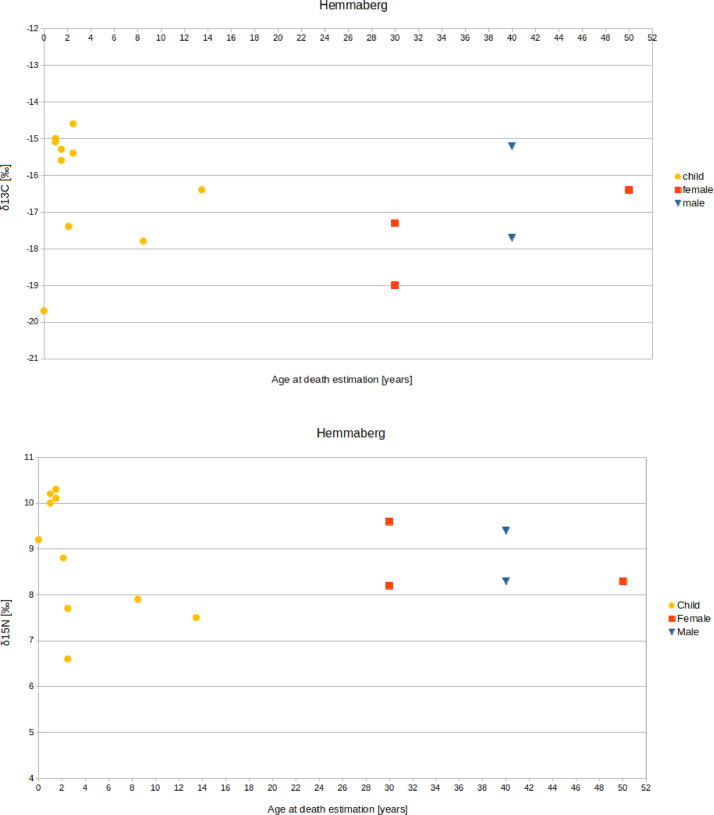
Result of the isotope analysis - (A) δ^13^C and (B) δ^15^N values - in relation to age and sex of the examined individuals from Hemmaberg/Gora svete Heme.

**Fig. 5:**
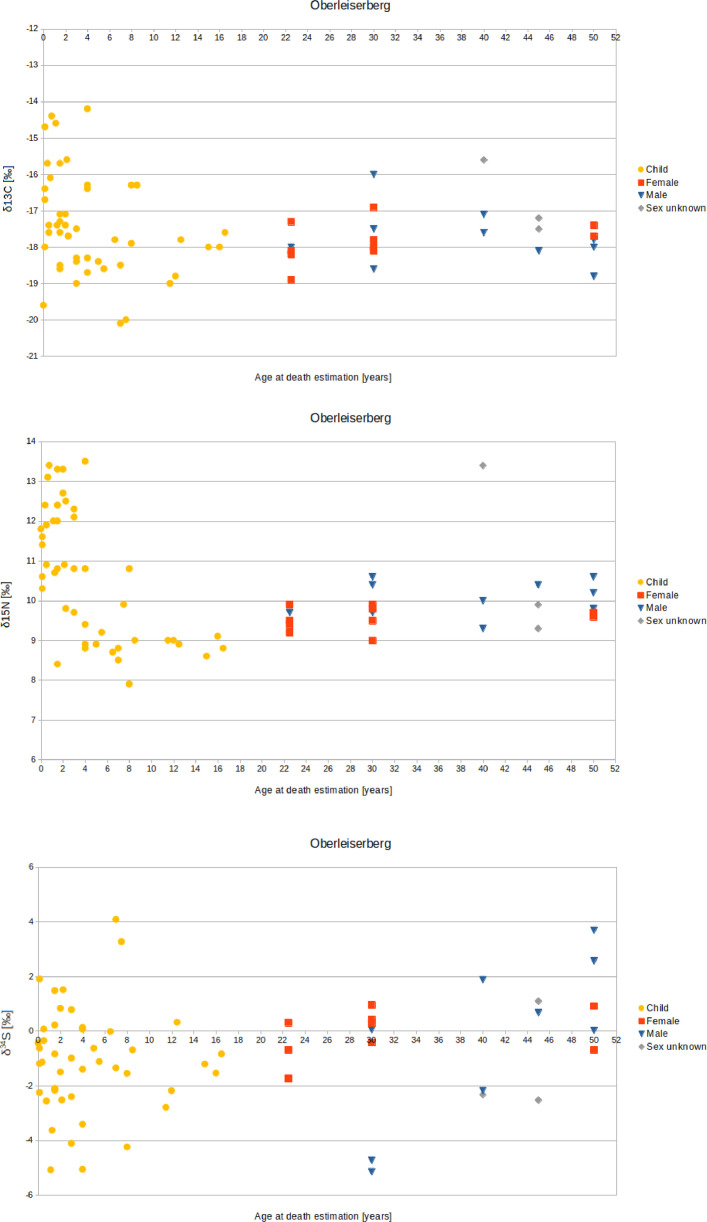
Result of the isotope analysis - (A) δ^13^C, (B) δ^15^N, and (C) δ^34^S values - in relation to age and sex of the examined individuals from Oberleiserberg.

The Oberleiserberg individuals have a mean δ^13^C value of –17.5 ± 1.2‰ (1σ), a mean δ^15^N value of +10.4 ± 1.5‰ (1σ), and a mean δ^34^S value of –0.9 ± 2.0‰ (1σ). For adults, the mean δ^13^C value is –17.7 ± 0.8‰ (1σ), the mean δ^15^N value +10.0 ± 0.9‰ (1σ), and the mean δ^34^S value is –0.4 ± 2.1‰ (1σ) (females: –17.9 ± 0.6‰, +9.6 ± 0.3‰, and –0.2 ± 1‰, respectively; males: –17.7 ± 0.8‰, +10.1 ± 0.4‰, and –0.4 ± 3.1‰, respectively). For subadults, the mean δ^13^C value is –17.4 ± 1.4‰ (1σ), the mean δ^15^N value is +10.6 ± 1.7‰ (1σ), and the mean δ^34^S value is –1.1 ± 1.9‰ (1σ) (Fig. 3, Fig. 4, Fig. 5).

## 3 Experimental Design, Materials and Methods

### 3.1 Mutualization of Isotopic and Anthropological Data Compilation Efforts in Austria Between THANADOS and IsoArcH

THANADOS (https://thanados.net [2]) and IsoArcH (www.isoarch.eu [1]) are two big data initiatives with complementary objectives. While THANADOS collates data from archaeologically and anthropologically analyzed burial grounds, IsoArcH gathers data from isotopically investigated bioarchaeological samples from all time periods and locations around the world. Thus, both initiatives collect a wide range of metadata on their materials and topics of interest, which overlap in some aspects. Case in point, archaeothanatological information or stable isotope results may be overlapped in both databases. THANADOS and IsoArcH have therefore agreed to pool their compilation efforts in the area of today's Austria. Data sharing is currently done through submission forms, before greater data interoperability between each database's systems is implemented and automatizes the process. To date, two archaeological sites are subject to such data exchange: i.e. the cemeteries of Hemmaberg/Gora svete Heme and Oberleiserberg.

### 3.2 Presentation of the Archaeological Sites

Located atop a limestone cliff in the Weinviertel region (Lower Austria, Austria), the Oberleiserberg cemetery contains graves that date back to the first half of the 11^th^ century [7]. Archaeological excavations have revealed the presence of 71 graves containing 77 individuals. Seven additional graves were uncovered in the 1930s [7]; those graves have been excluded from this study as their findings and documentation have been lost. With its mixture of Arpardian Age and Eastern Alpine objects, the cemetery is situated between the different spheres of influence of the consolidating Babenberg Margraviate, the Hungarian Kingdom, and the Přemyslids [7].

The Hemmaberg/Gora svete Heme cemetery is located in the foothills of the Karawanks, just west of Globasnitz (Carinthia, Austria). Although the site is recognized for its late antique/migration period center of pilgrimage featuring four churches, an early medieval burial ground was uncovered to the north of the complex. The church of SS Hemma and Dorothea, which is still in use today, is also located in the same vicinity. 29 individuals were buried in 28 graves. An additional burial was dated to the modern period by AMS radiocarbon dating [8].

The results of the anthropological analyses as well as the isotopic values have been initially published in open-access on https://thanados.net [2]. For more information on the sites visit Hemmaberg/Gora svete Heme and Oberleiserberg on https://thanados.net [2]).

### 3.3 Bone Collagen Extraction and Stable Isotope Analyses For Oberleiserberg

All individuals from the cemetery were sampled. Primarily, ribs were used. If ribs were not present, cranial bones (burial 41a to 41c, burial without find number 5) and in one case a metacarpal bone (burial without find number 1), were sampled. The Isotope Laboratory at Simon Fraser University assigned unique and consecutive S-SFU numbers to all samples, and for Oberleiserberg specifically, the samples were given numbers from S-SFU 178 to 253.

Extraction of collagen was performed according to the specifications in Richards and Hedges [3], supplemented by additional ultrafiltration of the sample material as recommended by Brown et al. [4].

The bones were cleaned and sampled using a Dremel 8200. Each bone sample was weighed and treated with 0.5 M hydrochloric acid (HCl), then stored in the refrigerator until digestion was complete. If needed, specific samples were transferred to fresh HCl to continue the process, and left at room temperature to shorten the reaction time. The prepared samples underwent cleaning using ultrapure water, followed by solubilization in hydrochloric acid with a pH of 3. After sealing the samples in test tubes, they were heated in a block at 75°C for 48 hours until complete dissolution. Next, the samples underwent filtration using Ezee filters followed with 30kDA Pall Microsep Centrifuge filters. The filtered collagen samples were freeze-dried for 48 hours. Finally, the samples were weighed and sent Iso-Analytical laboratory in Crewe (UK), where they were analyzed in the isotope abundance mass spectrometer with a coupled elemental analyzer (Elemental Analysis - Isotope Ratio Mass Spectrometry, EA-IRMS).

The criteria applied and the associated limit values for determining the quality of the extracted collagen are displayed in Table 2. These serve to rule out post-depositional diagenesis.

**Tab. 2:** Summary of collagen quality criteria and accepted value ranges (Ambrose and Norr [9], DeNiro [10], Harbeck and Grupe [11], van Klinken [12], Grupe et al. [13], Nehlich and Richards [14]).

Collagen Quality Criteria	Accepted Range
%Collagen	0.5 – 22 %
%C	15.3 – 47 %
%N	5.5 – 17.3 %
%S (for mammals)	0.15-0.35 %
C:N	2.9 – 3.6
C:S (for mammals)	600 ± 300
N:S (for mammals)	200 ± 100

Regarding collagen content, the samples from Oberleiserberg displayed values between 0.8 and 16.5 %. Two of the samples tested - individual 13 (0 %) and 53 (0.48 %) - did not meet the minimum requirements for collagen content.

Three other samples - individual 42 (too high values for carbon and nitrogen), individual 57 and finding number 3100 (both samples with too low values for sulfur) did not meet the collagen quality criteria.

### 3.4 Bone Collagen Extraction and Stable Isotope Analyses For Hemmaberg/Gora Svete Heme

From the early medieval cemetery of Hemmaberg/Gora svete Heme, 15 samples were analyzed. All samples were ribs or rib fragments. The bone samples were submitted to the Poznan Radiocarbon Laboratory for evaluation. Here each bone was checked for its carbon and nitrogen content by using the ThermoFischer NC analyzer before collagen was extracted [15]. Bones with a nitrogen content below 0.6% as well as those without sufficiently low C:N ratios (higher than 5) were excluded from further analyzes [15,16]. All bones submitted met the quality criteria and were integrated into the analysis.

Subsequently, collagen was extracted by using the method described by Longin [5] with additional alkali treatment as recommended by Piotrowska and Goslar [6]. Bones were crushed mechanically, and then treated with 2M HCl at room temperature for 20 minutes and 0.1M NaOH at room temperature for 1 hour. After each step the samples were centrifuged and residues were collected. The extraction of collagen was processed in HCl (pH=3, 80°C, 10h). After centrifugation the residues were removed. The extracted collagen was ultrafiltered by using precleaned Vivaspin 15 MWCO 30 kD filters [15], which removes broken amino acid chains of degraded collagen fraction [16].

The Poznan Radiocarbon Laboratory uses the C:N atomic ratio, which must fall between 2.7 and 3.5, and a minimum acceptable collagen extraction yield of 0.5%, as criteria for assessing collagen quality.

Samples that met the quality criteria were subsequently sent to the laboratory of the Goethe University in Frankfurt/Main. The C and N isotope measurements were performed on bulk organic matter using a Flash Elemental Analyzer 1112 (ThermoScientific), connected to a continuous flow inlet of a MAT 253 gas source mass spectrometer (Thermo Scientific).

Analytical precision is usually better than ±0.2‰ for δ^13^C and ±0.3‰ for δ^15^N. Two-point corrections are performed both for calibrating δ^13^C and δ^15^N values, running USGS 24, IAEA-CH-7, IAEA-N1 and IAEA-N2 along with the samples (personal information, T. Goslar, Poznan Radiocarbon Laboratory).

## Anthropological Data

While Burial 6 of the Hemmaberg/Gora svete Heme cemetery was investigated and published by Michaela Binder [8], the remaining burials of both cemeteries were anthropologically analyzed by Nina Richards [7]. The following research methods were used:

*Sex estimation*: Anthropological sex estimation was conducted macroscopically and according to Ferembach et al [17]. Determination of sex for subadult individuals is associated with high uncertainties and was not performed in the context of this study.

• *Age estimation*: Individuals were divided into the following age groups:

**Table:** 

• Fetus:	4^th^ lunar months to birth
• Neonate:	Birth to 3 months
• Infans 1:	3 months to 6.9 years
• Infans 2:	7 years to 13.9 years
• Juvenile:	14 to 19.9 years
• Adult:	20 to 39.9 years
• Mature:	40 to 59.9 years
• Senile:	60 years and older

Individuals that could not be categorized into one of these age groups due to bone preservation were categorized as subadults (0 to 19.9 years) or adults (20 years and older).

The methods used to determine age for subadult as well as adult individuals are similar to the methods stated in Brundke et al. [7].

## Ethics Statements

This work does not involve any experimentation on human and animal subjects.

## CRediT authorship contribution statement

**Nina Richards:** Investigation, Conceptualization, Data curation, Methodology, Writing – original draft, Visualization, Software, Funding acquisition. **Stefan Eichert:** Conceptualization, Data curation, Writing – review & editing, Software, Funding acquisition. **Sabine Ladstätter:** Investigation, Resources, Writing – review & editing. **Christina Cheung:** Investigation, Writing – review & editing. **Michael P. Richards:** Investigation, Resources, Writing – review & editing. **Kévin Salesse:** Conceptualization, Data curation, Writing – original draft, Visualization, Software.

## Declaration of Competing Interests

The authors declare that they have no known competing financial interests or personal relationships that could have appeared to influence the work reported in this paper.

## Data Availability

When big data initiatives meet: Data sharing between THANADOS and IsoArcH for early medieval cemeteries in Austria (Original data) (IsoArcH). Oberleiserberg (Original data) (THANADOS). Hemmaberg (Original data) (THANADOS)
